# Occupational exposures and coronary heart disease in the Hamburg City Health Study (HCHS) – a cross-sectional study

**DOI:** 10.1186/s12889-024-21259-1

**Published:** 2025-01-16

**Authors:** Franziska Labe, Raphael Twerenbold, Betül Toprak, Peter Koch, Birgit-Christiane Zyriax, Sarah Affolderbach, Lukas Damerau, Hanno Hoven, Hajo Zeeb, Robert Herold, Volker Harth

**Affiliations:** 1 Institute for Occupational and Maritime Medicine (ZfAM), University Medical Center Hamburg-Eppendorf (UKE), Hamburg, Germany; 2 University Center of Cardiovascular Science, Department of Cardiology, University Heart and Vascular Center Hamburg, University Medical Center Hamburg-Eppendorf (UKE), Hamburg, Germany; 3 German Center for Cardiovascular Research (DZHK) Partner Site Hamburg–Kiel–Lübeck, Hamburg, Germany; 4 Institute of Health Services Research in Dermatology and Nursing (IVDP), University Medical Center Hamburg-Eppendorf (UKE), Hamburg, Germany; 5 Midwifery Science-Health Services Research and Prevention, Institute for Health Services Research in Dermatology and Nursing (IVDP), University Medical Center Hamburg-Eppendorf (UKE), Hamburg, Germany; 6 Department of Prevention and Evaluation, Leibniz Institute for Prevention Research and Epidemiology - BIPS, Bremen, Germany; 7 Health Sciences Bremen, University of Bremen, Bremen, Germany

**Keywords:** Coronary heart disease, Ischemic heart disease, Myocardial infarction, Occupation, Occupational exposure, Job exposure matrix, Hamburg City Health Study

## Abstract

**Background:**

Coronary heart disease (CHD) is the leading cause of death among adults in Germany. There is evidence that occupational exposure to particulate matter, noise, psychosocial stressors, shift work and high physical workload are associated with CHD. The aim of this study is to identify occupations that are associated with CHD and to elaborate on occupational exposures associated with CHD by using the job exposure matrix (JEM) BAuA-JEM ETB 2018 in a German study population.

**Methods:**

Cross-sectional data from 8,070 participants, members of the first sub-cohort of the Hamburg City Health Study (HCHS), was used. To classify occupations, we rely on standard occupational titles (ISCO-08). The level of exposure is assigned to each job using a JEM. CHD is measured by self-reported diagnosis. Absolute and relative frequencies were calculated. Using logistic regression, the association of CHD and standard occupation titles via ISCO-08 and the association of CHD and occupational exposures via JEM were calculated and adjusted for potentially confounding covariates. Multiple imputations with chained equations (MICEs) were applied for missing values. Sensitivity analyses were performed.

**Results:**

The CHD prevalence in the study population was 4.6% (95% CI 4.2–5.1). Occupations associated with CHD were *Physical and Engineering Science Technicians*, *Other Health Associate Professionals*,* General Office Clerks*,* Secretaries (general)*,* Material Recording and Transport Clerks*,* Hairdressers*,* Beauticians and Related Workers*,* Electronics and Telecommunications Installers and Repairers*,* Other Craft and Related Workers*,* Car*,* Van and Motorcycle Drivers*,* Mobile Plant Operators* and *Domestic*,* Hotel and Office Cleaners and Helpers*. Among occupational exposures retrieved from the JEM, *Environmental Demands* showed an association with CHD in the crude model but not after adjustment. The results remained robust in sensitivity analyses.

**Conclusions:**

This study is the first to assess the association of a wide range of occupations and occupational exposures with CHD in a German study population. We found no association between occupational exposures and CHD after adjustment, but 11 occupations associated with CHD were identified. The results are limited by cross-sectional design, healthy worker effect (HWE), and small group sizes. Further studies with a larger sample and longitudinal design containing data on occupational history, occupational exposures and time of CHD diagnosis are needed.

**Supplementary Information:**

The online version contains supplementary material available at 10.1186/s12889-024-21259-1.

## Background

Cardiovascular diseases (CVD) account for the largest share of all disease-related causes of death in Germany with 36% in 2018 [1], specifically led by coronary heart disease (CHD) [2]. The lifetime prevalence of CHD in the age of 45 to 64 is 6.4% in men and 3.6% in women, increasing to 16.5% (men) and 9.2% (women) in the age of 65 to 79 [3]. Risk factors for CHD confirmed in numerous studies are smoking [4, 5], physical inactivity [6, 7], unfavorable dietary habits [8, 9], obesity [10], hypertension [11], unfavorable cholesterol [12], diabetes mellitus [13], advanced age [14], male sex [14], and genetic predispositions [15]. Socio-economic disadvantage [16], stress such as major life events or financial stress [17] as well as environmental factors such as traffic noise [18] can also be associated with CHD.

Apart from sociodemographic and lifestyle factors, the role of occupation is discussed in several studies. For example, there was evidence for an increased CHD mortality among supermarket meat and deli workers in an US American population [19] and an increased risk for unfavorable cholesterol and hypertension, possibly leading to CHD, among male professional drivers in Serbia [20]. Regarding CVD in general, an increased stroke risk among drivers, civil engineers, cargo workers, manual workers as well as in the food and drink preparation or fishery industry was identified in a Japanese population [21].

While studies about specific occupations associated with CHD are rare, there are several studies focusing on the underlying occupational exposures associated with CHD, some of them using job exposure matrices (JEMs) to link exposure profiles to surveyed occupations if exposure has not been measured directly. A review of 27 cohort studies clearly indicates an association of psychosocial stressors at work with CHD, especially regarding high job strain, long working hours, low job control and effort-reward imbalance [22–28]. Occupational exposure to particulate matter, especially through pesticides, urban air pollution, motor exhaust and welding fumes was associated with CHD in several systematic reviews [29–32]. There are contradicting studies about the association between occupational noise and CHD. A systematic review with meta-analysis by Skogstad et al. [33] in 2016, as well as several other observational studies [34–37] indicated an association of occupational noise with various CVD but not distinctly with CHD. Opposed to that, recent studies including a systematic review by Anfossi et al. [22] as well as a survey by Michaud et al. [38] could not clearly associate occupational noise with CVD. Several systematic reviews and meta-analyses provided strong evidence for an association of shift work and CHD [22, 39–41]. This association was found to be amplified when accompanied by other occupational exposures such as noise and physical workload [34, 42, 43]. Studies addressing the sole association of occupational physical workload and CHD led to inconclusive results [42–46]. Cold environment could also be identified as an occupational risk factor for CHD [35].

While there are already several studies addressing the association of occupational exposures and CHD, in Germany, no studies analysing a broad range of occupational exposures and occupational factors in the general working population have been conducted so far. The few existing German studies, some of them only focusing on CVD in general and not distinctly CHD, mainly consist of male populations [47–52] and mostly apply to specific industry branches such as asphalt workers [47], carbon black workers [48], chemical workers [49] and uranium miners [50, 51]. In addition to that, most studies are potentially outdated due to constantly changing working conditions [37, 47, 48, 50–52]. Regarding studies about specific occupations associated with CHD there were no comparable German studies found either.

The methodological approach of this study is guided by the work of De Matteis et al. [53, 54] and Sadhra et al. [55]. In the context of chronic obstructive pulmonary disease (COPD), these researchers initially examined the associations between various occupational groups and COPD. In subsequent studies, they explored the underlying occupational exposures using a job-exposure matrix (JEM). Similarly, the aim of this explorative study is to identify occupations that are associated with CHD and to elaborate on a broad spectrum of occupational exposures associated with CHD using a JEM in a German cohort.

## Materials and methods

### Study design and setting

Data basis was the Hamburg City Health Study (HCHS), a single-center population-based cohort study including randomly selected subjects aged 45 to 74 years at inclusion from the official population registry of Hamburg, Germany [56]. The study is registered at clinicaltrials.gov on 2019-01-04 (NCT03934957). For this study, cross-sectional data of the first sub-cohort containing 10,000 participants from 2016 to 2019 was used. To be included in this study, participants must have provided information about past diagnoses of CHD as well as about their current occupation, or if not employed at the time of survey, their last occupation. The remaining analysis population consisted of 8,070 participants.

### Assessment of CHD

CHD was defined as self-reported lifetime prevalence of a medical diagnosis of coronary heart disease, as assessed in the course of a medical history interview during the baseline examination for the HCHS. Myocardial infarction (MI) can result from CHD and is classified within the same category as CHD in the International Classification of Diseases (ICD-10). However, since not all cases of MI are caused by CHD and self-reported MI may be limited by non-specific symptoms, such as chest pain [57], self-reported medical diagnoses of MI were not included in the CHD definition in this study.

### Assessment of occupation and occupational exposures

Occupation was surveyed through several free-text answers that are subsequently classified into occupational codes with the International Standard Classification of Occupations 2008 (ISCO-08) [58]. ISCO-08 consists of 436 4-digit codes that can be grouped in 130 3-digit codes, 43 2-digit codes or 10 1-digit codes. This article focused on the 3-digit codes in order to achieve detailed information about occupational groups while still maintaining reasonably large group sizes.

Occupational exposures were operationalized by linking the ISCO-08 codes with the BAuA-JEM ETB 2018; a JEM by the Federal Institute for Vocational Education and Training (BIBB) and the Federal Institute for Occupational Safety and Health (BAuA), based on data of the BIBB/BAuA Employment Survey (ETB) 2018 in Germany [59]. As for occupational groups, the BAuA-JEM ETB 2018 was applied based on the ISCO-08 3-digit codes. The additional gain of knowledge of the 4-digit codes in comparison to the 3-digit codes was estimated to be low [59].

The BAuA-JEM ETB 2018 comprises the five work-related exposure groups, each containing specific items that are dichotomized based on the observed frequency or occurrence in each ISCO-08 occupational group: ***Autonomy*** (three items: *ability to plan and organize own work; ability to influence the amount of work; ability to decide when to take a break*), ***Work Intensity*** (six items: *overchallenged by workload; deadline/performance pressure; being disturbed/interrupted at work; performing different tasks or processes at the same time; go to the limits of one’s capabilities; having to work very quickly*), ***Physical Demands*** (five items: *working in a standing position; working in a sitting position for at least one hour without interruption; performing work requiring great manual dexterity; lifting and carrying heavy loads; working in forced positions*), ***Environmental Demands*** (five items: *working in smoky or dusty conditions or under gases and vapors; working in conditions of cold*,* heat*,* wet*,* moisture or draughts; working with oil*,* fat*,* dirt and filth; working in bright or insufficient lighting; working in noisy conditions*) and ***Working Time Location*** (three items: *work on Saturdays (at least once a month); work on Sundays (at least once a month); working time beyond 07:00–19:00*). The authors of the JEM originally included ***Social Support*** (seven items: *feel part of a community; good collaborations; support from colleagues; support from direct supervisor; praise/recognition from direct supervisor; never: not being informed in time about far-reaching decisions; never: not receiving necessary information about the job in time*) as an additional category. However, only a maximum of 1% of the variation in the construct could be attributed to differences between occupations. Consequently, this category was not considered further and was excluded from the JEM [59].

Each ISCO-08 occupational code is assigned a decile value per exposure group, which is derived from z-standardized sum values of the individual items in the respective exposure group. Occupational groups in the first decile represent the 10% of occupations with the lowest job demands, while those in the 10th decile represent the highest. The JEM uses linear multilevel models with random intercepts to adjust for gender, age, working hours and length of employment. The specific items per exposure group as well as further information about the JEM are described in detail by Meyer et al. [59]. Due to lack of data, the BAuA-JEM ETB 2018 does not provide decile values for some ISCO-08 codes. For this study, these cases (*n* = 11 in 3-digit coded groups) were imputed using data of the next-higher digit-group. Specifically, there was no information on ISCO-08 codes 223 (*Traditional and Complementary Medicine Professionals*, *n* = 8) and 835 (*Ships’ Deck Crews and Related Workers*, *n* = 3), which were imputed using ISCO-08 codes 22 (*Health Professionals*) and 83 (*Drivers and Mobile Plant Operators*), respectively.

### Assessment of adjustment covariates

#### Sociodemographic factors

Sex and age at time of the interview were retrieved from the Population Registration Office. Education was classified in low, middle and high based on the International Standard Classification of Education (ISCED) 2011 [60]. Type of employment was assessed by self-administered questionnaire and was divided into fulltime employment, part-time employment, other employment (partial retirement, marginal employment, irregular employment, vocational training, retraining, voluntary service or parental leave) and no current employment.

#### Health related factors

Body Mass Index (BMI) was categorized into underweight (< 18.5 kg/m²), normal weight (18.5–24.9 kg/m²), overweight (25–29.9 kg/m²) and obesity (≥ 30 kg/m²) based on height and weight measurement in the course of the study [61]. Hypertension was defined as either a measured resting systolic blood pressure of > 140 mmHg, a measured resting diastolic blood pressure of > 90 mmHg, the use of antihypertensive medication or self-reported medical diagnosis of hypertension [62]. Diabetes mellitus was defined as self-reported lifetime prevalence of a medical diagnosis, surveyed in the course of a medical history interview. Unfavorable cholesterol was defined by either measured non-High-Density-Lipoprotein (non-HDL) cholesterol of ≥ 146 mg/dL or the use of lipid-lowering medication [63].

#### Lifestyle factors

Dietary pattern was measured by a modified food-frequency-questionnaire based on the adherence score to Dietary Approaches to Stop Hypertension (DASH) [64] divided in approximate quartiles (≤ 3.5 points, < 3.5 - ≤ 4.5 points, < 4.5 - ≤ 5.0 points, > 5.0 points). Sport was divided into never performing any sport and performing any sport. Smoking was categorized as never, formerly (at least six months ago) and currently smoking. Dietary pattern, sport and smoking were measured by self-administered questionnaire.

### Statistical analyses

All statistical analyses were performed in the statistical software R (version 4.2.2). Absolute and relative frequencies were calculated and multiple binary logistic regression analyses were performed. Crude odds ratios (ORs) as well as adjusted odds ratios (AORs) and 95% confidence intervals (CIs) were calculated.

For the models measuring the association of occupations and CHD, the ISCO-08 1-digit group *Professionals* was selected as reference category based on large group size and low proportion of CHD cases (see Table 1). To maintain a sufficiently big group size in the 3-digit-based models, the reference group was not further divided. Estimates are only reported for groups with at least ten participants and three CHD cases.

The models were adjusted by sex, type of employment, education and age. This set of adjustment variables was extracted from a hypothetical directed acyclic graph (DAG) that was created based on causal relationships between potentially relevant covariates retrieved from the literature (additional Fig. 1). It contains the minimal sufficient adjustment set for the total effect between occupations and CHD. As sensitivity analysis, another adjustment set containing the following covariates was applied: sex, type of employment, education, age, dietary pattern, sport, unfavorable cholesterol, hypertension, BMI, diabetes mellitus, smoking. This set includes a wide range of potentially relevant covariates retrieved from the literature. Other potential confounders, such as non-work-related stress or environmental factors were not measured in this study and therefore remain unaccounted for. For numeric covariates, the assumption of linearity of logits was tested using Box-Tidwell and the assumption of no multicollinearity present was considered to be met if variance inflation factors (VIFs) < 10 [65]. Akaike information criterions (AICs) were calculated for model comparison.

Missing values in adjustment covariates were imputed via multiple imputations with chained equations (MICEs) [66], based on ten imputations and ten iterations. Missing values in ISCO-08 and CHD variables were addressed by listwise exclusion. The AICs of MICE imputed models were calculated as mean AIC of all imputations. A stratified analysis was conducted to examine potential differences between those who are currently employed and those who are not. For sensitivity analyses the population was reduced to participants who had worked at least five years in their stated occupation. This aimed to reduce the potential bias associated with recent job changes, especially after time of diagnosis.

## Results

### Description of the study population

**Fig. 1:**
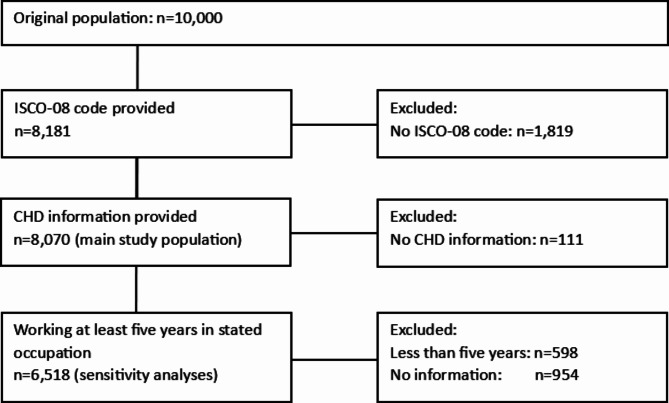
Flowchart of main study population and population of sensitivity analyses

Figure 1 shows the flowchart of the study population. The original population of 10,000 was reduced to the main study population of 8,070 due to exclusion of participants who did not provide information about their current or last occupation or CHD diagnosis. Of the 1,819 participants who did not provide information on their occupation (mean age: 66.6 years, 55.6% female), 127 cases of CHD were reported (*n* = 1,654 without CHD, *n* = 38 with no CHD information). The restricted population for sensitivity analyses (min. of 5 years in stated occupation) included 6,518 participants.

In the main study population of 8,070 participants, the CHD prevalence was 4.6% (95% CI 4.2–5.1) with 371 cases. The prevalence of CHD including MI amounted to 5.4% (95% CI: 5.0–6.0) with 439 cases. Table 1 shows the description of the main study population stratified by CHD diagnosis. The age in the main study population ranged from 46 to 78 years with a mean of 61.4 years. The mean age of participants with CHD diagnosis was higher than for participants with no CHD diagnosis (67.4 years, 61.1 years, respectively). Sex was equally distributed in the main study population, although among CHD cases there were 78.2% males and only 21.8% females. Equal parts of the participants had a middle or high education (48.7%, 47.6%, respectively), while only 3.7% had a low education. Among all participants, 41.3% worked full-time, 15.3% part-time, 6.9% in other types of employment (partial retirement, marginal employment, irregular employment, vocational training, retraining, voluntary service or parental leave) and 36.5% were not employed at the time of the survey. Among the 2,810 non-employed participants, 2,392 were pensioners, 143 were housewives or househusbands, 87 were jobseekers, 41 were permanently unable to work, 2 were students, 50 were categorized as “other” and 95 had missing data. Of the 2,392 pensioners, 40% had been retired for less than five years, and an additional 27% had been retired for less than 10 years. The largest share of jobseekers (41%) had been unemployed for less than one year. Among CHD cases, the biggest share of participants was not employed at the time of the survey (60.8%).

The most frequently represented occupational groups according to the ISCO-08 1-digit code were *Professionals* (29.4%), *Technicians and Associate Professionals* (24.3%) and *Clerical Support Workers* (17.4%). The highest CHD prevalences occurred in *Plant and Machine Operators and Assemblers* with 8.5% (18 CHD cases among 212 individuals) and *Craft and Related Trades Workers* with 7.9% (36 CHD cases among 453 individuals), the lowest in *Armed Forces Occupations* with 0% (0 CHD cases among 10 individuals) and *Professionals* with 3.6% (86 CHD cases among 2,369 individuals).

**Table 1:** Description of main study population (*n* = 8,070) stratified by diagnosis of CHD

	No CHD (*n* = 7,699)		CHD (*n* = 371)		Total (*n* = 8,070)	
Characteristic (valid answers)	Mean	SD	Mean	SD	Mean	SD
Age [years] (*n* = 8,070)	61.1	8.3	67.4	6.7	61.4	8.3
**Characteristic (valid answers)**	**n**	**%**	**n**	**%**	**n**	**%**
Sex (*n* = 8,070)						
Male	3,738	48.6	290	78.2	4,028	49.9
Female	3,961	51.4	81	21.8	4,042	50.1
Education (*n* = 8,019)						
Low	279	3.6	17	4.6	296	3.7
Middle	3,725	48.7	178	48.1	3,903	48.7
High	3,645	47.7	175	47.3	3,820	47.6
Type of employment (*n* = 7,704)						
Full-time	3,102	42.2	81	23.0	3,183	41.3
Part-time	1,157	15.7	20	5.7	1,177	15.3
Other	497	6.8	37	10.5	534	6.9
No employment	2,596	35.3	214	60.8	2,810	36.5
Unfavorable cholesterol (*n* = 7,712)						
No	2,985	40.6	43	11.7	3,028	39.3
Yes	4,361	59.4	323	88.3	4,684	60.7
Hypertension (*n* = 7,699)						
No	2,790	38.1	21	5.7	2,811	36.5
Yes	4,541	61.9	347	94.3	4,888	63.5
Diabetes mellitus (*n* = 8,050)						
No	7,287	94.9	305	82.7	7,592	94.3
Yes	394	5.1	64	17.3	458	5.7
BMI (*n* = 7,635)						
Underweight (< 18.5 kg/m²)	81	1.1	0	0.0	81	1.1
Normal weight (18.5–24.9 kg/m²)	2,908	40.0	82	22.9	2,990	39.2
Overweight (25–29.9 kg/m²)	2,914	40.0	169	47.2	3,083	40.4
Obesity (≥ 30 kg/m²)	1,374	18.9	107	29.9	1,481	19.4
Smoking (*n* = 8,055)						
Current	1,555	20.2	56	15.2	1,611	20.0
Former	3,339	43.4	231	62.6	3,570	44.3
Never	2,792	36.3	82	22.2	2,874	35.7
Dietary pattern (DASH score) (*n* = 7,454)						
≤ 3.5 points	1,794	25.2	95	28.2	1,889	25.3
> 3.5 - ≤ 4.5 points	2,381	33.5	113	33.5	2,494	33.5
> 4.5 - ≤ 5.0 points	1,186	16.7	58	17.2	1,244	16.7
> 5.0 points	1,756	24.7	71	21.2	1,827	24.5
Sports (*n* = 7,559)						
Not performing any sports	1,913	26.5	119	34.7	2,032	26.9
Performing any sports	5,203	73.5	224	65.3	5,527	73.1
ISCO-08 1-digit code (*n* = 8,070)						
0 Armed Forces Occupations	10	0.1	0	0.0	10	0.1
1 Managers	718	9.3	43	11.6	761	9.4
2 Professionals	2,283	29.7	86	23.2	2,369	29.4
3 Technicians and Associate Professionals	1,878	24.4	85	22.9	1,963	24.3
4 Clerical Support Workers	1,338	17.4	68	18.3	1,406	17.4
5 Services and Sales Workers	670	8.7	27	7.3	697	8.6
6 Skilled Agricultural, Forestry and Fishery Workers	19	0.2	1	0.3	20	0.2
7 Craft and Related Trades Workers	417	5.4	36	9.7	453	5.6
8 Plant and Machine Operators and Assemblers	194	2.5	18	4.9	212	2.6
9 Elementary Occupations	172	2.2	7	1.9	179	2.2

Abbreviations: BMI, Body Mass Index; CHD, Coronary Heart Disease; DASH, Dietary Approaches to Stop Hypertension; ISCO-08, International Standard Classification of Occupations 2008; SD, Standard Deviation

### Occupations and CHD via ISCO-08

Table 2 shows the crude, minimally and fully adjusted association of occupations measured via ISCO-08 and CHD in the main study population of 8070 participants. Results are displayed by 3-digit ISCO-08 code order. The AICs were 3065.6 for the crude model, 2760 for the minimally adjusted model and 2552.8 for the fully adjusted model. The assumptions of linearity of logits and no multicollinearity were met for all analyses of this article.

**Table 2:** Association of occupations and coronary heart disease (*n* = 8,070)

ISCO-08 3-digit occupational code and description			Crude model		Minimally adjusted model^1^**		Fully adjusted model^2^**	
	*n* / cases		OR	95% CI	OR	95% CI	OR	95% CI
2 Professionals (*Reference*)*	2,369	86	1	**-**	1	-	1	-
112 Managing Directors and Chief Executives	260	16	1.74	(0.97–2.94)	1.40	(0.80–2.47)	1.39	(0.78–2.51)
121 Business Services and Administration Managers	139	10	2.06	(0.98–3.87)	1.54	(0.76–3.11)	1.36	(0.66–2.81)
122 Sales, Marketing and Development Managers	47	3	1.81	(0.43–5.09)	1.78	(0.51–6.20)	1.95	(0.54–7.01)
132 Manufacturing, Mining, Construction and Distribution Managers	69	7	**3.00**	**(1.22–6.32)**	2.26	(0.97–5.28)	2.24	(0.90–5.58)
134 Professional Services Managers	117	4	0.94	(0.28–2.30)	0.84	(0.29–2.38)	0.77	(0.27–2.23)
311 Physical and Engineering Science Technicians	215	17	**2.28**	**(1.29–3.82)**	**2.12**	**(1.20–3.73)**	**1.92**	**(1.07–3.44)**
312 Mining, Manufacturing and Construction Supervisors	107	7	1.86	(0.76–3.85)	1.28	(0.56–2.90)	1.24	(0.53–2.90)
315 Ship and Aircraft Controllers and Technicians	24	3	3.79	(0.88–11.27)	1.86	(0.53–6.62)	1.37	(0.37–5.09)
322 Nursing and Midwifery Associate Professionals	197	7	0.98	(0.41–2.00)	2.19	(0.95–5.07)	1.68	(0.72–3.95)
325 Other Health Associate Professionals	109	6	1.55	(0.59–3.34)	**3.27**	**(1.32–8.11)**	**3.40**	**(1.34–8.65)**
331 Financial and Mathematical Associate Professionals	231	8	0.95	(0.42–1.87)	1.46	(0.67–3.16)	1.39	(0.63–3.06)
332 Sales and Purchasing Agents and Brokers	106	7	1.88	(0.77–3.89)	1.74	(0.76–4.01)	1.46	(0.63–3.41)
333 Business Services Agents	111	3	0.74	(0.18–2.01)	0.94	(0.29–3.11)	0.86	(0.26–2.88)
334 Administrative and Specialized Secretaries	363	10	0.75	(0.36–1.39)	1.17	(0.58–2.36)	1.13	(0.55–2.31)
335 Government Regulatory Associate Professionals	194	10	1.44	(0.69–2.69)	1.30	(0.65–2.60)	1.18	(0.58–2.41)
343 Artistic Cultural and Culinary Associate Professionals	52	3	1.63	(0.39–4.54)	2.30	(0.67–7.89)	2.58	(0.72–9.25)
411 General Office Clerks	289	18	**1.76**	**(1.01–2.91)**	**2.37**	**(1.33–4.24)**	**2.09**	**(1.15–3.79)**
412 Secretaries (general)	442	16	1.00	(0.56–1.67)	**2.13**	**(1.16–3.91)**	**1.91**	**(1.02–3.57)**
421 Tellers, Money Collectors and Related Clerks	104	3	0.79	(0.19–2.15)	0.96	(0.29–3.24)	0.79	(0.23–2.75)
431 Numerical Clerks	186	9	1.35	(0.62–2.59)	1.86	(0.88–3.95)	1.68	(0.78–3.63)
432 Material Recording and Transport Clerks	164	14	**2.48**	**(1.32–4.33)**	**2.49**	**(1.31–4.73)**	**2.29**	**(1.18–4.45)**
441 Other Clerical Support Workers	145	7	1.35	(0.56–2.77)	2.02	(0.87–4.69)	1.84	(0.78–4.34)
514 Hairdressers, Beauticians and Related Workers	47	5	**3.16**	**(1.07–7.49)**	**6.25**	**(2.25–17.35)**	**5.62**	**(1.90–16.61)**
522 Shop Salespersons	363	15	1.14	(0.63–1.94)	1.40	(0.77–2.57)	1.23	(0.66–2.29)
712 Building Finishers and Related Trades Workers	45	4	2.59	(0.77–6.60)	1.88	(0.63–5.62)	1.21	(0.38–3.82)
721 Sheet and Structural Metal Workers, Moulders and Welders and Related Workers	26	3	3.46	(0.81–10.19)	2.13	(0.59–7.70)	1.57	(0.42–5.94)
741 Electrical Equipment Installers and Repairers	57	6	**3.12**	**(1.17–6.94)**	2.48	(0.99–6.22)	2.38	(0.91–6.24)
742 Electronics and Telecommunications Installers and Repairers	49	7	**4.42**	**(1.78–9.55)**	**2.89**	**(1.19–7.01)**	**2.66**	**(1.06–6.67)**
754 Other Craft and Related Workers	21	4	**6.25**	**(1.77–17.30)**	**8.66**	**(2.56–29.33)**	**7.89**	**(1.95–31.92)**
832 Car, Van and Motorcycle Drivers	58	8	**4.25**	**(1.82–8.76)**	**3.49**	**(1.52–8.01)**	**2.63**	**(1.09–6.33)**
833 Heavy Truck and Bus Drivers	71	4	1.58	(0.47–3.94)	1.56	(0.53–4.58)	1.20	(0.40–3.61)
834 Mobile Plant Operators	11	3	**9.95**	**(2.15–35.09)**	**8.74**	**(2.00–38.29)**	**11.54**	**(2.33–57.22)**
911 Domestic, Hotel and Office Cleaners and Helpers	69	5	2.07	(0.71–4.81)	**4.21**	**(1.50–11.80)**	2.75	(0.92–8.26)

Note: Groups with less than 10 participants and/or less than 3 cases are not displayed. Statistically significant values in bold

Abbreviations: CI, Confidence Interval; ISCO-08, International Standard Classification of Occupations 2008; OR, Odds Ratio

* Reference category includes all participants of ISCO-08 1-digit code 2: *Professionals*

^1^ Adjusted by sex, type of employment, education, age

^2^ Adjusted by sex, type of employment, education, age, dietary pattern, sport, unfavorable cholesterol, hypertension, BMI, diabetes mellitus, smoking

** Imputed covariates via multiple imputations with chained equations (MICEs)

Occupations showing an association with CHD in the minimally adjusted model were ISCO-08 codes *311 (Physical and Engineering Science Technicians*, AOR_1_ 2.12 [95% CI 1.20–3.73]), *325 (Other Health Associate Professionals*, AOR_1_ 3.27 [95% CI 1.32–8.11]), *411* (*General Office Clerks*, AOR_1_ 2.37 [95% CI 1.33–4.24]), *412 (Secretaries (general)*, AOR_1_ 2.13 [95% CI 1.16–3.91]), *432 (Material Recording and Transport Clerks*, AOR_1_ 2.49 [95% CI 1.31–4.73]), *514 (Hairdressers*,* Beauticians and Related Workers*, AOR_1_ 6.25 [95% CI 2.25–17.35]), *742 (Electronics and Telecommunications Installers and Repairers*, AOR_1_ 2.89 [95% CI 1.19–7.01]), *754 (Other Craft and Related Workers*, AOR_1_ 8.66 [95% CI 2.56–29.33]), *832 (Car*,* Van and Motorcycle Drivers*, AOR_1_ 3.49 [95% CI 1.52–8.01]), *834 (Mobile Plant Operators*, AOR_1_ 8.74 [95% CI 2.00–38.29]) and *911 (Domestic*,* Hotel and Office Cleaners and Helpers*, AOR_1_ 4.21 [95% CI 1.50–2.33–11.80]).

In the stratified analysis, ISCO codes 742 (*Electronics and Telecommunications Installers and Repairers*) and 832 (*Car*,* Van*,* and Motorcycle Drivers*) were associated with CHD in the minimally adjusted model for both employed and unemployed participants. Additionally, among the unemployed participants, the following ISCO codes were associated with CHD: 112 (*Managing Directors and Chief Executives*), 121 (*Business Services and Administration Managers*), 311 (*Physical and Engineering Science Technicians*), 432 (*Material Recording and Transport Clerks*), and 721 (*Sheet and Structural Metal Workers*,* Moulders*,* Welders*,* and Related Workers*), *see additional* Table 1.

### Occupational exposures and CHD via BAuA-JEM ETB 2018

Table 3 displays the association of the BAuA-JEM ETB 2018 exposure groups based on the ISCO-08 3-digit codes with imputed decile values. *Environmental Demands* were associated with CHD in the crude model (OR 1.20 [95% CI 1.09–2.32]) but did not remain statistically significant after adjustment. The other occupational exposure groups did not show any association. The AICs for the crude, minimally and fully adjusted models were 3,004.0, 2,698.4 and 2,488.8, respectively, indicating best model fit for the fully adjusted model. The mean values of the exposure groups ranged between 4.7 and 6.0 (data not shown).

**Table 3:** Association of occupational exposure groups and coronary heart disease (*n* = 8,070)

Occupational exposure group	Crude model		Minimally adjusted model^1^*		Fully adjusted model^2^*	
	OR	95% CI	OR	95% CI	OR	95% CI
Autonomy	1.07	(1.00–1.14)	1.01	(0.94–1.08)	1.00	(0.93–1.08)
Work Intensity	0.98	(0.94–1.02)	1.01	(0.97–1.05)	1.01	(0.97–1.06)
Physical Demands	0.92	(0.84–1.02)	0.97	(0.88–1.07)	0.99	(0.90–1.10)
Environmental Demands	**1.20**	**(1.09–1.32)**	1.08	(0.98–1.19)	1.04	(0.94–1.15)
Working Time Location	0.97	(0.92–1.03)	0.97	(0.92–1.03)	0.97	(0.91–1.03)

Note: Statistically significant values in bold

Abbreviations: CI, Confidence Interval; OR, Odds Ratio

^1^ Adjusted by sex, type of employment, education, age

^2^ Adjusted by sex, type of employment, education, age, dietary pattern, sport, unfavorable cholesterol, hypertension, BMI, diabetes mellitus, smoking

* Imputed covariates via multiple imputations with chained equations (MICEs)

In the stratified analysis, no associations between occupational exposures and CHD were observed among the employed participants. However, among the unemployed participants, both *Autonomy* and *Environmental Demands* were associated with CHD, see additional Table 2.

### Occupations and occupational exposures

Table 4 shows the occupational exposure decile values derived from the BAuA-JEM ETB 2018 of occupations that presented an association with CHD in the minimally adjusted model. The exposure groups Physical Demands and Environmental Demands showed the highest mean decile values among selected occupations (6.9 and 7.0, respectively) while Autonomy and Work Intensity showed the lowest mean decile values (4.0 and 3.5, respectively). Among the selected occupations, Hairdressers, Beauticians and Related Workers and Domestic, Hotel and Office Cleaners and Helpers showed the highest mean decile values of the exposure groups with 7.2 and 6.6, respectively. General Office Clerks and Secretaries (general) showed the lowest mean decile values with 4.4 and 4.2, respectively. Comparing the mean decile values of the 11 selected occupations with those of the total study population without the selected occupations, higher demands were observed for *Physical Demands* (6.9 vs. 4.7), *Environmental Demands* (7.0 vs. 4.6), and *Working Time Location* (5.9 vs. 5.1).

**Table 4:** Occupational exposure decile values derived from BAuA-JEM ETB 2018 of selected occupations

ISCO-08 3-digit code and description	Autonomy	Work Intensity	Physical Demands	Environ-mental Demands	Working Time Location	Mean
311 Physical and Engineering Science Technicians	5	2	5	7	4	4.6
325 Other Health Associate Professionals	3	7	8	6	6	6.0
411 General Office Clerks	6	4	4	4	4	4.4
412 Secretaries (general)	6	4	4	3	4	4.2
432 Material Recording and Transport Clerks	4	7	5	6	5	5.4
514 Hairdressers, Beauticians and Related Workers	5	6	9	8	8	7.2
742 Electronics and Telecommunications Installers and Repairers	5	1	9	8	3	5.2
754 Other Craft and Related Workers	3	2	9	8	7	5.8
832 Car, Van and Motorcycle Drivers	1	1	6	8	9	5.0
834 Mobile Plant Operators	1	3	7	10	8	5.8
911 Domestic, Hotel and Office Cleaners and Helpers	5	2	10	9	7	6.6
**Mean decile values of selected occupations**	4.0	3.5	6.9	7.0	5.9	-
**Mean decile values and SD of the total study population without selected occupations** (***n*** **=** **6**,**596))**	6.2 (SD 2.9)	6.1 (SD 2.7)	4.7 (SD 2.8)	4.6 (SD 2.9)	5.1 (SD 3.0)	-

Abbreviations: ISCO-08, International Standard Classification of Occupations 2008; SD, Standard Deviation

### Sensitivity analyses

After exclusion of participants working less than five years in their stated occupation, the following differences compared to the main analysis appeared in the minimally adjusted model: *Electrical Equipment Installers and Repairers* (AOR_1_ 3.17 [95% CI 1.23–8.17]) showed an association with CHD, while *Car*,* Van and Motorcycle Drivers* and *Mobile Plant Operators* were not associated with CHD anymore in this model. The estimates were similar but slightly higher in this sensitivity analysis in comparison to the main analysis. The AICs of the crude, minimally and fully adjusted models were 2,466.4, 2,210.7 and 2,075.6, respectively.

Regarding occupational exposures and CHD, the following differences appeared: In the crude model, *Autonomy* (OR 1.11 [95% CI 1.02–1.19]) was associated with CHD additionally to *Environmental Demands* (OR 1.15 [95% CI 1.03–1.28]). As in the main analysis, there was no association after adjustment. The AICs were 2,394.4, 2,146.3 and 2,006.7 for the crude, minimally and fully adjusted model. The mean values of the exposure groups ranged between 4.6 and 6.0 (data not shown).

Estimates of the crude, minimally adjusted and fully adjusted associations of occupations and CHD as well as occupational exposures and CHD for this population can be viewed in additional Tables 3 and 4.

## Discussion

This research aimed to identify occupational exposures and occupations associated with CHD in a German population. While several occupations were found to be associated with CHD, there was no conclusive association between occupational exposures and CHD via JEM.

In the main study population, the self-reported lifetime prevalence of CHD was 4.6% (males 7.2%, females 2.0%). For comparison, the German Health Update (GEDA 2019/2020-EHIS) showed a CHD prevalence of 5.8% (males 6.4%, females 3.6%) for the general German population aged 45 to 64 years [3]. The slight underestimation of prevalence in the present study population compared to the GEDA 2019/2020-EHIS data could be explained by misclassification due to self-reported diagnosis and by different CHD case definitions as for example GEDA includes MI in the CHD definition [3]. Regional health disparities might also explain the observed differences as Hamburg shows a lower CHD prevalence with 4.0% in comparison to the general German population [3]. Selection bias through recruitment of comparably healthy and well-educated subjects (participation bias) or through death by CHD of potential study subjects before time of recruitment may also have affected the surveyed prevalence.

### Occupations and CHD via ISCO-08

Occupations associated with CHD in this study were *Physical and Engineering Science Technicians*,* Other Health Associate Professionals*,* General Office Clerks*,* Secretaries (general)*,* Material Recording and Transport Clerks*,* Hairdressers*,* Beauticians and Related Workers*,* Electronics and Telecommunications Installers and Repairers*,* Other Craft and Related Workers*,* Car*,* Van and Motorcycle Drivers*,* Mobile Plant Operators* and *Domestic*,* Hotel and Office Cleaners and Helpers*.

A recently published paper of the German Gutenberg Health Study showed an association of the occupational dimension of socioeconomic status with CVD [16]. Regarding the association of a wide range of occupations with CHD in Germany there are no comparable studies. A comparative study from Tüchsen et al. [67], based on data from Denmark, Great Britain and Italy from the 1970s to 1990s, supports some of results found in this study as they identified an association with CHD mortality in warehouse and wholesale staff, barbers and hairdressers, radio and telegraph operators and drivers. A Canadian retrospective study from Finkelstein et al. [68], based on data before 2000, identified an increased CHD mortality among heavy equipment operators, which supports the association of *Mobile Plant Operators* and CHD found in this study. The found association of *Car*,* Van and Motorcycle Drivers* and CHD is supported by the studies of Djindjić et al. [20] and Fukai et al. [21], although their found associations were not focused on CHD but other CVD. Fukai et al. [21] also observed an association of stroke with civil engineers, cargo workers and manual workers, which can be applied to the found associations of CHD with *Physical and Engineering Science Technicians*,* Mobile Plant Operators* and *Other Craft and Related Workers*. This study cannot find an association of people in the food and fishery industry and CHD, which is contradicting to the findings of Fukai et al. [21] and Jadhav et al. [19]. As all of the named studies are either not directly addressing CHD or are based on outdated data or data from different countries, the comparability is limited. Also, the different classification systems of occupations applied in this article and in cited studies impedes the comparability.

As reference category, an ISCO-08 1-digit group was chosen to obtain a sufficiently large group. Compared to the other 1-digit groups, the group *Professionals* provided relatively low prevalence and high group size and therefore was chosen. Still, it included a wide range of occupations and contained 86 CHD cases. Further analyses in more extensive study populations with a more specific reference category are advisable. The wide CIs in some associations, especially among *Hairdressers*,* Beauticians and Related Workers*,* Other Craft and Related Workers* and *Mobile Plant Operators* are also likely to be caused by low group and case numbers, also calling for larger studies.

The results of the minimally adjusted model remained robust in comparison to the fully adjusted model. The AICs indicate a slightly better statistical fit of the fully adjusted model. However, the minimally sufficient adjustment set should generally provide a comparably more unbiased association of exposure and outcome (total effect), since the adjustment of covariates that are positioned on the assumed causal path of exposure and outcome in the DAG may introduce bias [69, 70]. Nevertheless, DAGs are built based on assumptions, so the derived minimal sufficient adjustment set may be incorrect, incomplete or only one of many possible sets [69]. Also, some covariates could either act as a confounder or a mediator or even both, which justifies both an adjustment and no adjustment by these covariates [71]. The fully adjusted model had a bigger share of MICE imputed data which might be seen as limitation. *Domestic*,* Hotel and Office Cleaners and Helpers* showed an association only in the minimally adjusted model, not in the fully adjusted model. This could be an indicator for adjusting for mediator bias in the fully adjusted model. Remarkably, the sensitivity analysis revealed that the association persists across all models. In further studies, detailed mediation analyses are advisable to understand the mechanisms and relations of utilized covariates.

Despite the wide range of adjustment variables applied on the models, unmeasured factors might still be present and influence the estimates. Especially factors as socioeconomic status, non-work-related stress and environmental factors could play a crucial role in the context of CHD. Therefore, the adjustment is potentially incomplete and the impact of confounding and mediation through those unmeasured factors cannot be estimated.

### Occupational exposures and CHD via BAuA-JEM ETB 2018

There was no association of occupational exposures and CHD found in the adjusted models, only the crude model showed an association of *Environmental Demands* and CHD, which was no longer significant after adjustment. The literature reports associations of particulate matter such as pesticides, urban air pollution, motor exhaust and welding fumes [29, 31, 32] as well as noise [34–36] with CHD, but they were weak in our study. Other occupational exposures mentioned in the literature such as shift work, physical workload and psychosocial factors could not be linked to CHD in this study either. However, a more detailed exposure assessment could help identify and target additional occupational exposures associated with CHD, including pesticides, urban air pollution, motor exhaust, welding fumes, noise, and shift work. To this end, specific JEMs designed for these exposures could be utilized.

In this study, the lack of associations between occupational exposures and CHD after adjustment might have been affected by the use of a JEM as a surrogate measure of occupational exposures. Specifically, the BAuA-JEM ETB 2018 provides a general assessment at the 3-digit ISCO level, which offers limited detail. Furthermore, the matrix was based on a general population job survey that relied on participants’ self-reports, which could introduce bias - particularly with respect to exposures that are poorly assessed by workers, such as chemical exposures. Due to the dichotomization of exposure group items and the formation of sum scores in the BAuA-JEM ETB 2018, initially complex data about diverse occupational exposures was strongly simplified, which could have limited the informative value of the generated results. Using JEM data generally implies the assumption that occupational exposures are homogenous within each occupation, which in reality might not always be the case. Bias may have also been introduced as the BAuA-JEM ETB 2018 was based on a different study population. However, the BAuA-JEM ETB 2018 relies on German population data from 2018 and the mean values of the occupational exposure groups in this population indicate that the distribution of occupational exposures in the HCHS population is similar to the population of the BAuA-JEM ETB 2018. Further studies with detailed data about occupational exposures are advisable to gain more insight on potentially undetected associations with CHD. Examining potential interactions between separate occupational exposures regarding CHD, as indicated by preceding studies [34, 42, 43], should also be a focus of further research.

### Occupations and occupational exposures

The examination of occupations associated with CHD in the minimally adjusted model regarding their decile values derived from the BAuA-JEM ETB 2018 exposure groups showed high values in *Environmental Demands* and *Physical Demands.* High values in *Environmental Demands* were expected due to the previously discussed analyses of this article as well as the literature, however *Physical Demands* did not show an association with CHD in our analyses. General physical activity is viewed as cardioprotective [6, 7, 72], while a positive association of occupational physical activity with CHD could be shown in some studies, but was inconclusive in others [42–45]. The potentially protective or detrimental effects of physical activity on CHD depending on the context is known as the occupational physical activity health paradox [44] and could have played a role in our findings.

Among the occupations associated with CHD in the minimally adjusted model, *General Office Clerks* and *Secretaries (general)* showed relatively low decile values in the BAuA-JEM ETB 2018 exposure groups. This could be an indicator for occupational exposure agents that were not covered by the BAuA-JEM ETB 2018. Also, selection bias may have appeared as people whose CHD had caused permanent health damages might have switched their occupation into one without high occupational exposures. For further studies, a breakdown of the specific items in each occupational exposure group of the BAuA-JEM ETB 2018 associated with CHD could generate more insights. The linkage of occupations showing an association with CHD in this study and the deciles derived from BAuA-JEM ETB 2018 is limited by the different data sources and can only raise hypotheses for further studies.

### Strengths and limitations

The results of this study are limited by the cross-sectional design as it is not ensured that CHD developed through the course of the known occupation. There cannot be drawn conclusions about causal relationships, only about associations. For this study population, there was no data available on the full occupational history so that exposures from past jobs remained unaccounted for. In the Hamburg City Health Study, detailed job histories were queried only for employees in shift work. In the present study, 1,283 participants reported shift work and provided information on CHD; however, this was insufficient for detailed analyses. Future studies should comprehensively account for occupational careers and document exposure by considering all jobs. This approach would enable the assignment of an exposure score that incorporates both the duration and level of exposure, provided the assessment methods allow for such analysis. As there was no data available about the time of CHD diagnosis there is no certainty about the chronology of exposure and outcome. The most common occupations in the study population were *Managers* (ISCO-08 1-digit code: 1, 9.4%), *Professionals* (group 2, 29.4%), *Technicians and Associate Professionals* (group 3, 24.3%), and *Clerical Support Workers* (group 4, 17.4%). Occupation groups 1 to 3 collectively comprised 63.1% of the study population. In comparison, a report from the German Federal Statistical Office stated that 46.6% of employed persons in Germany were in highly qualified occupations (groups 1 to 3) in 2022, with a share of 49.6% for females and 44.0% for males [73]. This comparison indicates that the study population is predominantly representative of individuals employed in highly qualified occupations.

The healthy worker effect (HWE), which is likely to occur in cross-sectional occupational health related studies, could have influenced the results, especially through healthy worker survivor effect [74]. The prevalence of unfavorable cholesterol levels, defined as either non-HDL cholesterol exceeding 146 mg/dL or the use of lipid-lowering medication, was 60.7% in the study population. Findings from the DEGS1 study on the health of German adults reported a prevalence of total cholesterol levels exceeding 190 mg/dL of 70.9% (95% CI: 68.1–73.6) in the 45–64 age group and 71.4% (95% CI: 68.4–74.3) in the 65–79 age group [74]. The prevalence of hypertension, defined as either a measured resting systolic blood pressure > 140 mmHg, a measured resting diastolic blood pressure > 90 mmHg, the use of antihypertensive medication, or a self-reported medical diagnosis of hypertension, was 63.5% in the study population. In comparison, a study on the 12-month prevalence of hypertension in Germany, defined as a self-reported medical diagnosis of hypertension or the use of antihypertensive medication, did not include measurements of exact blood pressure values. The reported prevalence of hypertension in that study was 31.6% (95% CI: 29.9–33.5) for females aged 45–64 years, 63.8% (95% CI: 61.5–66.1) for females aged 65 years and older, 38.3% (95% CI: 36.4–40.1) for males aged 45–64 years, and 65.1% (95% CI: 62.9–67.1) for males aged 65 years and older [75]. Despite all methodological differences compared to studies on the general German population, these results suggest that the present study population is relatively healthy.

CHD can cause severe health damages and carries a high mortality. As there was no data available about CHD mortality cases but only about self-reported lifetime prevalence of diagnosis, occupationally exposed persons who died from CHD before cohort inception could – by definition – not be taken into account. Participants might also have dropped out of employment or have changed into a different occupation due to health damages caused by CHD. This would have led to bias towards the null and an underestimation of associations. In further studies it could therefore be beneficial to examine the association of occupations and occupational exposures with preliminary states of CHD as for example atherosclerosis. As the extent of HWE is highly dependent on factors like sex, age, duration of employment and occupation itself, it is barely possible to eliminate the bias with certainty. The consideration of time under exposure via lifetime occupational histories or the internal comparison of different doses of occupational exposure agents would have been helpful to minimize the bias but there is currently no such data available for this study population. The adjustment for type of employment as well as the inclusion of currently not employed participants by taking into account their last occupation both aimed to minimize the HWE. Nevertheless, there is no certainty about the extent of the remaining bias through HWE and the results of this study should be interpreted with caution [76].

As already discussed, the use of the BAuA-JEM ETB 2018 may have introduced bias and also, misclassifications of occupational codes cannot be ruled out. A strength of the BAuA-JEM ETB 2018 on the other hand is that the occupational exposures of each group were calculated via multi-level models including the hierarchy of the ISCO-08 classifications (2 to 4 digits), adjusted by sex, age, working time and job tenure [59]. Therefore, potential bias through these factors has already been addressed in the process of the construction of the JEM.

Despite the relatively large study sample and the randomized selection of participants in the HCHS recruitment, representativeness of the sample cannot necessarily be assumed as selection bias may still occur, especially due to healthy worker survivor effect and single-center recruitment. The small proportion of participants with low education in this sample indicates sampling bias. External validity may be compromised. Due to the underrepresentation of participants with low education, an association of occupations with potentially precarious working conditions and CHD could remain undetected as low education and precarious working conditions are likely connected.

Despite the limitations the study also holds some strengths as it is the first study examining the association of a broad spectrum of occupations and occupational exposures with CHD in a recently recruited German population. Especially the identified occupations associated with CHD that remained robust in sensitivity analyses can be further used as starting point for more extensive and specific studies and for building target-group specific CHD prevention approaches.

Reproducing the analyses of this study in a more comprehensive study population regarding the included age range as well as the place of recruitment could also be beneficial, as the HCHS solely focuses on participants aged 45 to 74 from Hamburg. Extensive studies with longitudinal data including occupational histories as well as utilizing actual data on occupational exposures including duration and intensity of exposure are suggested. Future research could benefit from examining more detailed ISCO codes and a finer level of exposure assessment. While the 3-digit ISCO-08 codes used in this study are adequate for evaluating broad occupational exposures, they may not capture the nuances of specific occupational groups. For example, the group 325 (*Other Health Associate Professionals*) encompasses a range of occupations with differing exposures, such as 3251 (*Dental Assistants and Therapists*), 3254 (*Dispensing Opticians*), 3257 (*Environmental and Occupational Health Inspectors and Associates*), and 3258 (*Ambulance Workers*). A more granular analysis could provide valuable insights into these subgroups. Additionally, mediation analyses on covariates utilized in this study are suggested for further studies, as well as interaction analyses of exposure variables.

## Conclusion

This study identified several occupations associated with CHD while there could not be identified a conclusive association of occupational exposures and CHD via JEM. As this study is the first to assess the association of a wide range of occupations and occupational exposures with CHD in a German study population, there is a lack of comparable studies. The results of this study can provide a foundation for further research as other large German population-based studies such as NAKO and Gutenberg Health Study may be analysed in a similar way in the future. Regarding policy implications, after further validation of the findings of this study, identified occupations and related exposures should be focused on regarding occupational health and safety measures for the prevention of CHD in the future.

## Electronic supplementary material

Below is the link to the electronic supplementary material.

Supplementary Material 1

## Data Availability

The datasets used and analysed during the current study are available from the corresponding author on reasonable request.

## Abbreviations

AIC: Akaike Information Criterion
AOR: Adjusted Odds Ratio
BAuA: Federal Institute for Occupational Safety and Health
BIBB: Federal Institute for Vocational Education and Training
BMI: Body Mass Index
CHD: Coronary Heart Disease
CI: Confidence Interval
COPD: Chronic Obstructive Pulmonary Disease
CVD: Cardiovascular Disease
DAG: Directed Acyclic Graph
DASH: Dietary Approaches to Stop Hypertension
ETB: BIBB/BAuA Employment Survey
HCHS: Hamburg City Health Study
HDL: High-Density Lipoprotein
HWE: Healthy Worker Effect
ICD-10: International Classification of Diseases
ISCED: International Standard Classification of Education
ISCO-08: International Standard Classification of Occupations 2008
JEM: Job Exposure Matrix
MI: Myocardial Infarction
OR: Odds Ratio
SD: Standard Deviation
VIF: Variance Inflation Factor

## References

[1] Datenreport. 2021 - Ein Sozialbericht für die Bundesrepublik Deutschland. Bonn: Bundeszentrale für politische Bildung; 2021.

[2] Deaths caused by one. of the 10/20/50/100 most frequent causes of death (absolute, per 100,000, rank, percentage) (from 1998). Classification: years, region, age, sex, ICD-10 [https://www.gbe-bund.de/gbe/pkg_isgbe5.prc_menu_olap?p_uid=gastd&p_aid=99302335&p_sprache=E&p_help=2&p_indnr=516&p_version=1&p_ansnr=59883516].

[3] Dashboard zu Gesundheit in Deutschland aktuell - GEDA. 2019/2020 [https://public.tableau.com/app/profile/robert.koch.institut/viz/Gesundheit_in_Deutschland_aktuell/GEDA_20192020-EHIS]

[4] Chang CM, Corey CG, Rostron BL, Apelberg BJ. Systematic review of cigar smoking and all cause and smoking related mortality. BMC Public Health. 2015;15:390. 25907101 10.1186/s12889-015-1617-5 PMC4408600

[5] Hackshaw A, Morris JK, Boniface S, Tang J-L, Milenković D. Low cigarette consumption and risk of coronary heart disease and stroke: meta-analysis of 141 cohort studies in 55 study reports. BMJ (Clinical Res ed. 2018;360:j5855. 10.1136/bmj.j5855 PMC5781309 29367388

[6] Cleven L, Krell-Roesch J, Nigg CR, Woll A. The association between physical activity with incident obesity, coronary heart disease, diabetes and hypertension in adults: a systematic review of longitudinal studies published after 2012. BMC Public Health. 2020;20(1):726. 32429951 10.1186/s12889-020-08715-4 PMC7238737

[7] Kivimäki M, Singh-Manoux A, Pentti J, Sabia S, Nyberg ST, Alfredsson L, Goldberg M, Knutsson A, Koskenvuo M, Koskinen A, et al. Physical inactivity, cardiometabolic disease, and risk of dementia: an individual-participant meta-analysis. BMJ (Clinical Res ed). 2019;365:l1495. 10.1136/bmj.l1495 PMC6468884 30995986

[8] Aune D, Giovannucci E, Boffetta P, Fadnes LT, Keum N, Norat T, Greenwood DC, Riboli E, Vatten LJ, Tonstad S. Fruit and vegetable intake and the risk of cardiovascular disease, total cancer and all-cause mortality-a systematic review and dose-response meta-analysis of prospective studies. Int J Epidemiol. 2017;46(3):1029–56. 28338764 10.1093/ije/dyw319 PMC5837313

[9] Aune D, Keum N, Giovannucci E, Fadnes LT, Boffetta P, Greenwood DC, Tonstad S, Vatten LJ, Riboli E, Norat T. Whole grain consumption and risk of cardiovascular disease, cancer, and all cause and cause specific mortality: systematic review and dose-response meta-analysis of prospective studies. BMJ (Clinical Res ed). 2016;353:i2716. 10.1136/bmj.i2716 PMC4908315 27301975

[10] Mongraw-Chaffin ML, Peters SAE, Huxley RR, Woodward M. The sex-specific association between BMI and coronary heart disease: a systematic review and meta-analysis of 95 cohorts with 1·2 million participants. Lancet Diabetes Endocrinol. 2015;3(6):437–49. 25960160 10.1016/S2213-8587(15)00086-8 PMC4470268

[11] Stevens SL, Wood S, Koshiaris C, Law K, Glasziou P, Stevens RJ, McManus RJ. Blood pressure variability and cardiovascular disease: systematic review and meta-analysis. BMJ (Clinical Res ed). 2016;354:i4098. 10.1136/bmj.i4098 PMC4979357 27511067

[12] Liou L, Kaptoge S. Association of small, dense LDL-cholesterol concentration and lipoprotein particle characteristics with coronary heart disease: a systematic review and meta-analysis. PLoS ONE. 2020;15(11):e0241993. 33166340 10.1371/journal.pone.0241993 PMC7652325

[13] Cai X, Zhang Y, Li M, Wu JH, Mai L, Li J, Yang Y, Hu Y, Huang Y. Association between prediabetes and risk of all cause mortality and cardiovascular disease: updated meta-analysis. BMJ (Clinical Res ed. 2020;370:m2297. 10.1136/bmj.m2297 PMC7362233 32669282

[14] Robert K-I. Koronare Herzkrankheit. Faktenblatt zu GEDA 2012: Ergebnisse Der Studie »Gesundheit in Deutschland Aktuell 2012«. Berlin: RKI; 2014.

[15] Abraham G, Havulinna AS, Bhalala OG, Byars SG, Livera AM, Yetukuri L, Tikkanen E, Perola M, Schunkert H, Sijbrands EJ, et al. Genomic prediction of coronary heart disease. Eur Heart J. 2016;37(43):3267–78. 27655226 10.1093/eurheartj/ehw450 PMC5146693

[16] Hahad O, Gilan DA, Chalabi J, Al-Kindi S, Schuster AK, Wicke F, Büttner M, Tüscher O, Lackner KJ, Galle PR, et al. Cumulative social disadvantage and cardiovascular disease burden and mortality. European Journal of Preventive Cardiology; 2023. 10.1093/eurjpc/zwad264 37721449

[17] Santosa A, Rosengren A, Ramasundarahettige C, Rangarajan S, Gulec S, Chifamba J, Lear SA, Poirier P, Yeates KE, Yusuf R, et al. Psychosocial risk factors and Cardiovascular Disease and Death in a Population-based Cohort from 21 Low-, Middle-, and high-income countries. JAMA Netw open. 2021;4(12):e2138920. 34910150 10.1001/jamanetworkopen.2021.38920 PMC8674745

[18] Banerjee D, Das PP, Foujdar A. Association between road traffic noise and prevalence of coronary heart disease. Environ Monit Assess. 2014;186(5):2885–93. 24374763 10.1007/s10661-013-3587-3

[19] Jadhav S, Chedjieu IP, Faramawi MF, Ndetan H, Fischbach L, Thapa S, Johnson ES. Non-cancer mortality in workers in the meat and delicatessen departments of supermarkets (1950–2006). Environ Res. 2015;142:155–60. 26160045 10.1016/j.envres.2015.06.030

[20] Djindjić N, Jovanović J, Djindjić B, Jovanović M, Pesić M, Jovanović JJ. Work stress related lipid disorders and arterial hypertension in professional drivers - a cross-sectional study. Vojnosanit Pregl. 2013;70(6):561–8. 23885522 10.2298/vsp1306561d

[21] Fukai K, Furuya Y, Nakazawa S, Kojimahara N, Hoshi K, Toyota A, Tatemichi M. A case control study of occupation and cardiovascular disease risk in Japanese men and women. Sci Rep. 2021;11(1):23983. 34907236 10.1038/s41598-021-03410-9 PMC8671491

[22] Moretti Anfossi C, Ahumada Muñoz M, Tobar Fredes C, Pérez Rojas F, Ross J, Head J, Britton A. Work exposures and Development of Cardiovascular diseases: a systematic review. Ann Work Expo Health. 2022;66(6):698–713. 35237787 10.1093/annweh/wxac004 PMC9250287

[23] Rugulies R, Framke E, Sørensen JK, Svane-Petersen AC, Alexanderson K, Bonde JP, Farrants K, Flachs EM, Magnusson Hanson LL, Nyberg ST, et al. Persistent and changing job strain and risk of coronary heart disease. A population-based cohort study of 1.6 million employees in Denmark. Scand J Work Environ Health. 2020;46(5):498–507. 32202306 10.5271/sjweh.3891 PMC7737794

[24] Torén K, Schiöler L, Giang WK, Novak M, Söderberg M, Rosengren A. A longitudinal general population-based study of job strain and risk for coronary heart disease and stroke in Swedish men. BMJ Open. 2014;4(3):e004355. 24589825 10.1136/bmjopen-2013-004355 PMC3948640

[25] Selander J, Bluhm G, Nilsson M, Hallqvist J, Theorell T, Willix P, Pershagen G. Joint effects of job strain and road-traffic and occupational noise on myocardial infarction. Scand J Work Environ Health. 2013;39(2):195–203. 23032870 10.5271/sjweh.3324

[26] Kivimäki M, Kawachi I. Work stress as a risk factor for Cardiovascular Disease. Curr Cardiol Rep. 2015;17(9):630. 26238744 10.1007/s11886-015-0630-8 PMC4523692

[27] Pejtersen JH, Burr H, Hannerz H, Fishta A, Hurwitz Eller N. Update on work-related psychosocial factors and the development of ischemic heart disease: a systematic review. Cardiol Rev. 2015;23(2):94–8. 24979202 10.1097/CRD.0000000000000033

[28] Dragano N, Siegrist J, Nyberg ST, Lunau T, Fransson EI, Alfredsson L, Bjorner JB, Borritz M, Burr H, Erbel R, et al. Effort-reward imbalance at Work and Incident Coronary Heart Disease: a Multicohort Study of 90,164 individuals. Epidemiology. 2017;28(4):619–26. 28570388 10.1097/EDE.0000000000000666 PMC5457838

[29] Zago AM, Faria NMX, Fávero JL, Meucci RD, Woskie S, Fassa AG. Pesticide exposure and risk of cardiovascular disease: a systematic review. Glob Public Health 2020:1–23. 10.1080/17441692.2020.1808693 32816635

[30] Fang SC, Cassidy A, Christiani DC. A systematic review of occupational exposure to particulate matter and cardiovascular disease. Int J Environ Res Public Health. 2010;7(4):1773–806. 20617059 10.3390/ijerph7041773 PMC2872342

[31] De Marchis P, Verso MG, Tramuto F, Amodio E, Picciotto D. Ischemic cardiovascular disease in workers occupationally exposed to urban air pollution - A systematic review. Ann Agric Environ Med. 2018;25(1):162–6. 29575857 10.26444/aaem/79922

[32] Mocevic E, Kristiansen P, Bonde JP. Risk of ischemic heart disease following occupational exposure to welding fumes: a systematic review with meta-analysis. Int Arch Occup Environ Health. 2015;88(3):259–72. 25047981 10.1007/s00420-014-0965-2

[33] Skogstad M, Johannessen HA, Tynes T, Mehlum IS, Nordby KC, Lie A. Systematic review of the cardiovascular effects of occupational noise. Occup Med (Lond). 2016;66(1):10–6. 26732793 10.1093/occmed/kqv148

[34] Eriksson HP, Söderberg M, Neitzel RL, Torén K, Andersson E. Cardiovascular mortality in a Swedish cohort of female industrial workers exposed to noise and shift work. Int Arch Occup Environ Health. 2021;94(2):285–93. 32892225 10.1007/s00420-020-01574-x PMC7873009

[35] Pettersson H, Olsson D, Järvholm B. Occupational exposure to noise and cold environment and the risk of death due to myocardial infarction and stroke. Int Arch Occup Environ Health. 2020;93(5):571–5. 31915923 10.1007/s00420-019-01513-5 PMC7260257

[36] Kerns E, Masterson EA, Themann CL, Calvert GM. Cardiovascular conditions, hearing difficulty, and occupational noise exposure within US industries and occupations. Am J Ind Med. 2018;61(6):477–91. 29537072 10.1002/ajim.22833 PMC6897488

[37] Kersten N, Backé E. Occupational noise and myocardial infarction: considerations on the interrelation of noise with job demands. Noise Health. 2015;17(75):116–22. 25774615 10.4103/1463-1741.153403 PMC4918664

[38] Michaud DS, Marro L, McNamee JP. Self-reported occupational noise exposure and cardiovascular disease in Canada: results from the Canadian Health measures Survey. J Acoust Soc Am. 2021;150(2):990. 34470300 10.1121/10.0005588

[39] Wu QJ, Sun H, Wen ZY, Zhang M, Wang HY, He XH, Jiang YT, Zhao YH. Shift work and health outcomes: an umbrella review of systematic reviews and meta-analyses of epidemiological studies. J Clin Sleep Med. 2022;18(2):653–62. 34473048 10.5664/jcsm.9642 PMC8804985

[40] Torquati L, Mielke GI, Brown WJ, Kolbe-Alexander T. Shift work and the risk of cardiovascular disease. A systematic review and meta-analysis including dose-response relationship. Scand J Work Environ Health. 2018;44(3):229–38. 29247501 10.5271/sjweh.3700

[41] Cheng M, He H, Wang D, Xu L, Wang B, Ho KM, Chen W. Shift work and ischaemic heart disease: meta-analysis and dose-response relationship. Occup Med (Lond). 2019;69(3):182–8. 30923828 10.1093/occmed/kqz020

[42] Virkkunen H, Härmä M, Kauppinen T, Tenkanen L. The triad of shift work, occupational noise, and physical workload and risk of coronary heart disease. Occup Environ Med. 2006;63(6):378–86. 16709702 10.1136/oem.2005.022558 PMC2078113

[43] Virkkunen H, Härmä M, Kauppinen T, Tenkanen L. Shift work, occupational noise and physical workload with ensuing development of blood pressure and their joint effect on the risk of coronary heart disease. Scand J Work Environ Health. 2007;33(6):425–34. 18327510 10.5271/sjweh.1170

[44] Quinn TD, Yorio PL, Smith PM, Seo Y, Whitfield GP, Barone Gibbs B. Occupational physical activity and cardiovascular disease in the United States. Occup Environ Med. 2021;78(10):724–30. 33737330 10.1136/oemed-2020-106948

[45] Johnsen AM, Alfredsson L, Knutsson A, Westerholm PJ, Fransson EI. Association between occupational physical activity and myocardial infarction: a prospective cohort study. BMJ Open. 2016;6(10):e012692. 27697879 10.1136/bmjopen-2016-012692 PMC5073545

[46] Bonde JPE, Flachs EM, Madsen IE, Petersen SB, Andersen JH, Hansen J, Jørgensen EB, Kolstad H, Holtermann A, Schlünssen V, et al. Acute myocardial infarction in relation to physical activities at work: a nationwide follow-up study based on job-exposure matrices. Scand J Work Environ Health. 2020;46(3):268–77. 31725896 10.5271/sjweh.3863

[47] Burstyn I, Kromhout H, Partanen T, Svane O, Langård S, Ahrens W, Kauppinen T, Stücker I, Shaham J, Heederik D, et al. Polycyclic aromatic hydrocarbons and fatal ischemic heart disease. Epidemiology. 2005;16(6):744–50. 16222163 10.1097/01.ede.0000181310.65043.2f

[48] Morfeld P, Mundt KA, Dell LD, Sorahan T, McCunney RJ. Meta-analysis of Cardiac Mortality in three cohorts of Carbon Black Production Workers. Int J Environ Res Public Health 2016, 13(3). 10.3390/ijerph13030302 PMC4808965 27005647

[49] Yong M, Nasterlack M, Germann C, Lang S, Oberlinner C. Shift work and risk of non-cancer mortality in a cohort of German male chemical workers. Int Arch Occup Environ Health. 2014;87(7):763–73. 24297469 10.1007/s00420-013-0922-5

[50] Kreuzer M, Kreisheimer M, Kandel M, Schnelzer M, Tschense A, Grosche B. Mortality from cardiovascular diseases in the German uranium miners cohort study, 1946–1998. Radiat Environ Biophys. 2006;45(3):159–66. 16897062 10.1007/s00411-006-0056-1

[51] Gellissen J, Pattloch D, Möhner M. Effects of occupational exposure to respirable quartz dust on acute myocardial infarction. Occup Environ Med. 2019;76(6):370–5. 31010894 10.1136/oemed-2018-105540 PMC6585272

[52] Siegrist J, Peter R, Motz W, Strauer BE. The role of hypertension, left ventricular hypertrophy and psychosocial risks in cardiovascular disease: prospective evidence from blue-collar men. Eur Heart J. 1992;13(Suppl D):89–95. 1396866 10.1093/eurheartj/13.suppl_d.89

[53] De Matteis S, Jarvis D, Hutchings S, Darnton A, Fishwick D, Sadhra S, Rushton L, Cullinan P. Occupations associated with COPD risk in the large population-based UK Biobank cohort study. Occup Environ Med. 2016;73(6):378–84. 27001997 10.1136/oemed-2015-103406

[54] De Matteis S, Jarvis D, Darnton L, Consonni D, Kromhout H, Hutchings S, Sadhra SS, Fishwick D, Vermeulen R, Rushton L, et al. Lifetime occupational exposures and chronic obstructive pulmonary disease risk in the UK Biobank cohort. Thorax. 2022;77(10):997–1005. 35082144 10.1136/thoraxjnl-2020-216523

[55] Sadhra SSMN, Kurmi OP. Occupational exposure to inhaled pollutants and risk of airflow obstruction: a large UK population-based UK Biobank cohort. Thorax. 2020;75:468–75. 32376731 10.1136/thoraxjnl-2019-213407

[56] Jagodzinski A, Johansen C, Koch-Gromus U, Aarabi G, Adam G, Anders S, Augustin M, der Kellen RB, Beikler T, Behrendt C-A, et al. Rationale and design of the Hamburg City Health Study. Eur J Epidemiol. 2020;35(2):169–81. 31705407 10.1007/s10654-019-00577-4 PMC7125064

[57] Choe S, Lee J, Lee J, Kang D, Lee J-K, Shin A. Validity of self-reported stroke and myocardial infarction in Korea: the Health examinees (HEXA) study. J Prev Med Public Health. 2019;52(6):377–83. 31795614 10.3961/jpmph.19.089 PMC6893227

[58] International Standard Classification of Occupations. Structure, group definitions and correspondence tables. Online-Ausg edn. Volume 1. Geneva: International Labour Office; 2012.

[59] Meyer S-C, Siefer A. Development of a job-exposure matrix (JEM) based on the BIBB/BAuA Employment Survey 2018. Federal Institute for Occupational Safety and Health (BAuA); 2023.

[60] Statistics UIf. International standard classification of education: ISCED 2011. Comp Social Res 2012, 30.

[61] Defining Adult Overweight &, Obesity. [https://www.cdc.gov/obesity/basics/adult-defining.html]

[62] Hypertension. [https://www.who.int/news-room/fact-sheets/detail/hypertension]

[63] Mach F, Baigent C, Catapano AL, Koskinas KC, Casula M, Badimon L, Chapman MJ, De Backer GG, Delgado V, Ference BA, et al. 2019 ESC/EAS guidelines for the management of dyslipidaemias: lipid modification to reduce cardiovascular risk: the Task Force for the management of dyslipidaemias of the European Society of Cardiology (ESC) and European Atherosclerosis Society (EAS). Eur Heart J. 2020;41(1):111–88. 31504418 10.1093/eurheartj/ehz455

[64] Terschüren C, Damerau L, Petersen EL, Harth V, Augustin M, Zyriax BC. Association of Dietary Pattern, Lifestyle and Chronotype with Metabolic Syndrome in Elderly-lessons from the Population-based Hamburg City Health Study. Int J Environ Res Public Health 2021, 19(1). 10.3390/ijerph19010377 PMC8744785 35010639

[65] Eid M, Gollwitzer M, Schmitt M. Statistik und Forschungsmethoden: mit Online-Materialien, 4., überarbeitete und erweiterte Auflage edn. Weinheim; Basel: Beltz; 2015.

[66] Azur MJ, Stuart EA, Frangakis C, Leaf PJ. Multiple imputation by chained equations: what is it and how does it work? Int J Methods Psychiatr Res. 2011;20(1):40–9. 21499542 10.1002/mpr.329 PMC3074241

[67] Tüchsen F, Andersen O, Costa G, Filakti H, Marmot MG. Occupation and ischemic heart disease in the European Community: a comparative study of occupations at potential high risk. Am J Ind Med. 1996;30(4):407–14. 8892545 10.1002/(SICI)1097-0274(199610)30:4<407::AID-AJIM5>3.0.CO;2-S

[68] Finkelstein MM, Verma DK, Sahai D, Stefov E. Ischemic heart disease mortality among heavy equipment operators. Am J Ind Med. 2004;46(1):16–22. 15202121 10.1002/ajim.20036

[69] Foraita R, Spallek J, Zeeb H. Directed Acyclic Graphs. In: Handbook of Epidemiology. edn. Edited by Ahrens W, Pigeot I. New York, NY: Springer New York; 2014: 1481–1517.

[70] Tingting Wang HL, Ping S, Yu Y, Sun X, Liu Y, Yuan Z. Fuzhong Xue: sensitivity analysis for mistakenly adjusting for mediators in estimating total effect in observational studies. BMJ Open 2017, 7. 10.1136/bmjopen-2016-015640 PMC5719285 29162569

[71] VanderWeele TJ. Principles of confounder selection. Eur J Epidemiol. 2019;34(3):211–9. 30840181 10.1007/s10654-019-00494-6 PMC6447501

[72] Kraus WE, Powell KE, Haskell WL, Janz KF, Campbell WW, Jakicic JM, Troiano RP, Sprow K, Torres A, Piercy KL. Physical activity, all-cause and Cardiovascular Mortality, and Cardiovascular Disease. Med Sci Sports Exerc. 2019;51(6):1270–81. 31095084 10.1249/MSS.0000000000001939 PMC6527136

[73] Quality of employment -. Persons in employment in highly qualified occupations [https://www.destatis.de/EN/Themes/Labour/Labour-Market/Quality-Employment/Dimension6/6_1_PersonsEmploymentHighlyQualifiedOccupations.html]

[74] Scheidt-Nave C, Du Y, Knopf H, Schienkiewitz A, Ziese T, Nowossadeck E, Gößwald A, Busch MA. Verbreitung Von Fettstoffwechselstörungen Bei Erwachsenen in Deutschland. Bundesgesundheitsblatt - Gesundheitsforschung - Gesundheitsschutz. 2013;56(5):661–7. 23703484 10.1007/s00103-013-1670-0

[75] Neuhauser H, Kuhnert R, Born S. 12-Monats-Prävalenz Von Bluthochdruck in Deutschland. Volume 2. Robert Koch-Institut, Epidemiologie und Gesundheitsberichterstattung; 2017.

[76] Chowdhury R, Shah D, Payal AR. Healthy worker Effect Phenomenon: revisited with emphasis on statistical methods - a review. Indian J Occup Environ Med. 2017;21(1):2–8. 29391741 10.4103/ijoem.IJOEM_53_16 PMC5763838

