# Supplementary material for: Occupational exposures and coronary heart disease in the Hamburg City Health Study (HCHS) – a cross-sectional study
Source: BMC Public Health. 2025 Jan 16;25:180. doi: 10.1186/s12889-024-21259-1 (PMC11740511; doi:10.1186/s12889-024-21259-1)
Supplement: Supplementary file 1 — Supplementary Material 1 [file 12889_2024_21259_MOESM1_ESM.pdf]

Supplementary materials

Additional figure 1: Directed acyclic graph containing potentially relevant covariates retrieved from the literature

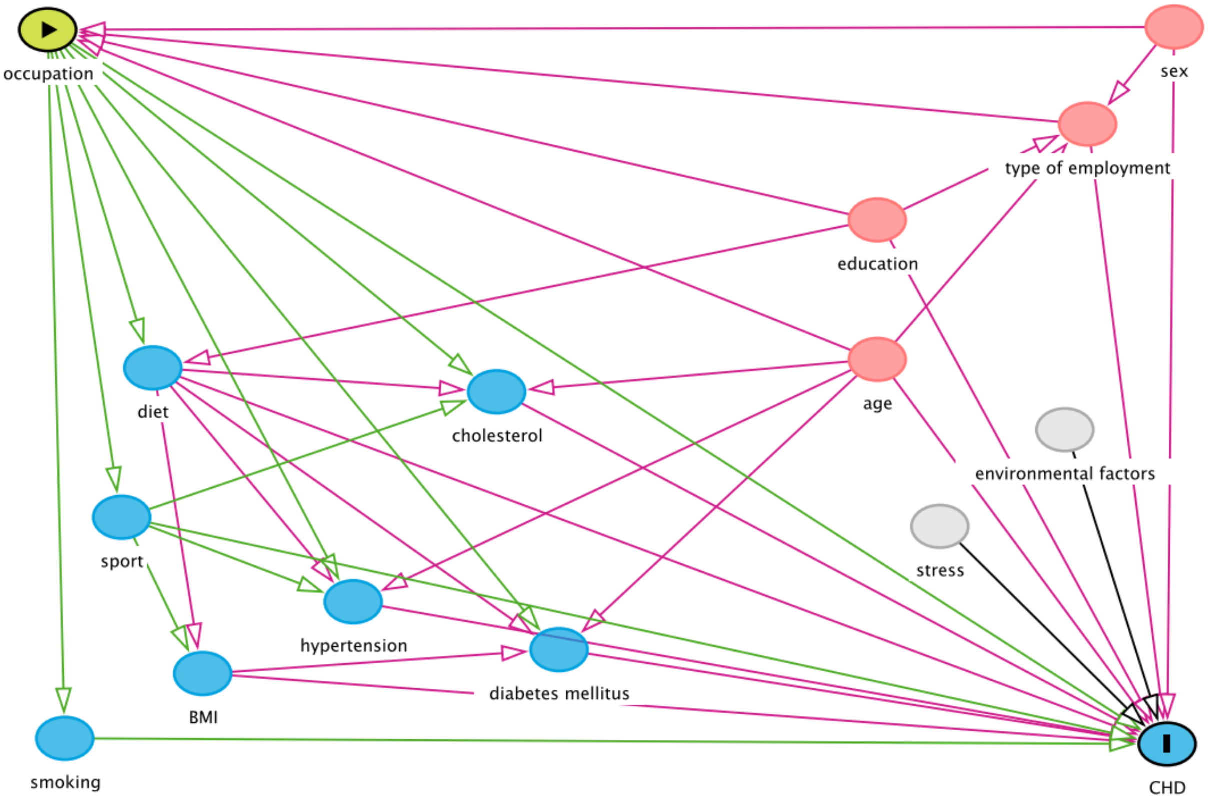

CHD: Coronary Heart Disease

Additional table 1: Association of occupations and coronary heart disease stratified by type of employment (n = 7,704)

ISCO-08 3-digit occupational code and description	Currently employed (n = 4,894) [1]				Currently unemployed (n = 2,810) [1]			
	n / cases		OR	95% CI	n / cases		OR	95% CI
2 Professionals (*reference*)*	1,521	40	1	-	762	44	1	-
112 Managing Directors and Chief Executives	-	-	-	-	82	10	2.3	(1.0 - 4.5)
121 Business Services and Administration Managers	-	-	-	-	55	8	2.8	(1.2 - 6.0))
311 Physical and Engineering Science Technicians	-	-	-	-	91	12	2.5	(1.2 - 4.8)
432 Material Recording and Transport Clerks	-	-	-	-	53	8	2.9	(1.2 - 6.2)
721 Sheet and Structural Metal Workers, Moulders and Welders and Related Workers	-	-	-	-	10	3	7.0	(1.5 - 26.1)
742 Electronics and Telecommunications Installers and Repairers	25	3	5.0	(1.2 - 15.3)	21	4	3.8	(1.1 - 10.9)
832 Car, Van and Motorcycle Drivers	42	4	3.9	(1.1 - 10.3)	12	3	5.4	(1.2 - 19.0)

Note: Only groups with statistically significant associations are shown.

Abbreviations: CI, Confidence Interval; ISCO-08, International Standard Classification of Occupations 2008; OR, Odds Ratio

* Reference category includes all participants of ISCO-08 1-digit code 2: Professionals

[1] Adjusted by sex, type of employment, education, age.

Additional table 2: Association of occupational exposure groups and coronary heart disease stratified by type of employment (n = 7,704)

Occupational exposure group	Currently employed (n = 4,894) [1]		Currently unemployed (n = 2,810) [1]	
	OR	95% CI	OR	95% CI
Autonomy	1.05	(0.94 - 1.18)	**1.10**	**(1.00 - 1.20)**
Work Intensity	0.99	(0.93 - 1.06)	0.97	(0.92 - 1.03)
Physical Demands	0.99	(0.84 - 1.16)	0.92	(0.81 – 1.03)
Environmental Demands	1.12	(0.96 – 1.30)	**1.20**	**(1.05 - 1.36)**
Working Time Location	0.97	(0.88 – 1.06)	1.00	(0.92 - 1.08)

Note: Statistically significant values in bold.

Abbreviations: CI, Confidence Interval; OR, Odds Ratio

[1] Adjusted by sex, type of employment, education, age.

Additional table 3: Association of occupations and coronary heart disease in participants who have worked at least five years in stated occupation (n=6,518)

ISCO-08 3-digit occupational code and description	n / cases		Crude model OR	95% CI	Minimally adjusted model [1,**] OR	95% CI	Fully adjusted Model [2,**] OR	95% CI
2 Professionals (*reference*)*	2,032	71	1	-	1	-	1	-
112 Managing Directors and Chief Executives	217	15	**2.05**	**(1.11 - 3.55)**	1.54	(0.85 - 2.79)	1.54	(0.83 - 2.84)
121 Business Services and Administration Managers	121	8	1.96	(0.85 - 3.93)	1.42	(0.65 - 3.11)	1.31	(0.59 - 2.92)
132 Manufacturing, Mining, Construction and Distribution Managers	59	5	2.56	(0.87 - 6.02)	1.89	(0.71 - 5.06)	1.92	(0.68 - 5.46)
134 Professional Services Managers	94	3	0.91	(0.22 - 2.51)	0.88	(0.26 - 2.92)	0.79	(0.23 - 2.70)
311 Physical and Engineering Science Technicians	174	14	**2.42**	**(1.28 - 4.26)**	**2.34**	**(1.25 - 4.37)**	**2.02**	**(1.06 - 3.84)**
312 Mining, Manufacturing and Construction Supervisors	92	4	1.26	(0.38 - 3.12)	0.88	(0.31 - 2.51)	0.87	(0.30 - 2.54)
322 Nursing and Midwifery Associate Professionals	147	3	0.58	(0.14 - 1.57)	1.55	(0.46 - 5.24)	1.18	(0.34 - 4.03)
325 Other Health Associate Professionals	82	5	1.79	(0.62 - 4.16)	**4.16**	**(1.50 - 11.49)**	**4.47**	**(1.58 - 12.63)**
331 Financial and Mathematical Associate Professionals	198	8	1.16	(0.51 - 2.31)	1.90	(0.86 - 4.19)	1.95	(0.87 - 4.37)
332 Sales and Purchasing Agents and Brokers	90	5	1.62	(0.56 - 3.75)	1.45	(0.55 - 3.83)	1.20	(0.45 - 3.21)
333 Business Services Agents	92	3	0.93	(0.22 - 2.56)	1.15	(0.34 - 3.86)	1.12	(0.33 - 3.81)
334 Administrative and Specialized Secretaries	286	6	0.59	(0.23 - 1.27)	0.98	(0.41 - 2.37)	0.99	(0.41 - 2.43)
335 Government Regulatory Associate Professionals	161	8	1.44	(0.63 - 2.88)	1.38	(0.63 - 3.01)	1.26	(0.57 - 2.81)
343 Artistic Cultural and Culinary Associate Professionals	47	3	1.88	(0.45 - 5.32)	2.57	(0.73 - 8.96)	2.71	(0.75 - 9.85)
411 General Office Clerks	227	14	1.82	(0.97 - 3.18)	**2.53**	**(1.31 - 4.89)**	**2.37**	**(1.21 - 4.64)**
412 Secretaries (general)	312	14	1.30	(0.69 - 2.26)	**2.88**	**(1.48 - 5.62)**	**2.62**	**(1.32 - 5.21)**
431 Numerical Clerks	145	7	1.40	(0.58 - 2.90)	2.16	(0.91 - 5.09)	1.92	(0.80 - 4.61)
432 Material Recording and Transport Clerks	134	12	**2.72**	**(1.37 - 4.97)**	**2.65**	**(1.31 - 5.36)**	**2.45**	**(1.19 - 5.05)**
441 Other Clerical Support Workers	107	5	1.35	(0.47 - 3.11)	2.29	(0.85 - 6.15)	1.88	(0.69 - 5.15)
514 Hairdressers, Beauticians and Related Workers	31	4	**4.09**	**(1.19 - 10.81)**	**8.24**	**(2.52 - 26.95)**	**7.21**	**(2.03 - 25.59)**
522 Shop Salespersons	277	12	1.25	(0.64 - 2.25)	1.41	(0.72 - 2.77)	1.33	(0.67 - 2.65)
712 Building Finishers and Related Trades Workers	40	4	3.07	(0.90 - 7.93)	2.26	(0.74 - 6.90)	1.48	(0.46 - 4.73)
741 Electrical Equipment Installers and Repairers	48	6	**3.95**	**(1.47 - 8.93)**	**3.17**	**(1.23 - 8.17)**	**3.26**	**(1.22 - 8.75)**
742 Electronics and Telecommunications Installers and Repairers	43	7	**5.37**	**(2.13 - 11.8)**	**3.46**	**(1.39 - 8.64)**	**3.35**	**(1.29 - 8.65)**
754 Other Craft and Related Workers	15	3	**6.90**	**(1.55 - 22.32)**	**9.33**	**(2.21 - 39.42)**	**8.46**	**(1.58 - 45.42)**
832 Car, Van and Motorcycle Drivers	37	4	3.35	(0.98 - 8.70)	2.64	(0.86 - 8.14)	2.13	(0.67 - 6.80)
833 Heavy Truck and Bus Drivers	53	3	1.66	(0.40 - 4.65)	1.80	(0.52 - 6.21)	1.36	(0.38 - 4.79)

911 Domestic, Hotel and Office Cleaners and Helpers	44	5	**3.54 (1.19 - 8.49)**	**9.51 (3.16 - 28.56)**	**6.17 (1.87 - 20.37)**

Note: Groups with less than 10 participants and/or less than 3 CHD cases are not displayed. Statistically significant values in bold.

Abbreviations: CI, Confidence Interval; ISCO-08, International Standard Classification of Occupations 2008; OR, Odds Ratio

* Reference category includes all participants of ISCO-08 1-digit code 2: Professionals

** Imputed covariates via multiple imputations with chained equations (MICEs)

[1] Adjusted by sex, type of employment, education, age

[2] Adjusted by sex, type of employment, education, age, dietary pattern, sport, unfavorable cholesterol, hypertension, BMI, diabetes mellitus, smoking

Additional table 4: Association of occupational exposure groups and coronary heart disease in participants who have worked at least five years in stated occupation (n=6,518)

	Crude model		Minimally adjusted model [1,*]		Fully adjusted model [2,*]	
**Occupational exposure group**	**OR**	**95% CI**	**OR**	**95% CI**	**OR**	**95% CI**
Autonomy	**1.11**	**(1.02 - 1.19)**	1.04	(0.96 - 1.12)	1.03	(0.95 - 1.12)
Work Intensity	0.97	(0.93 - 1.02)	1.00	(0.95 - 1.05)	1.00	(0.95 - 1.05)
Physical Demands	0.99	(0.88 - 1.10)	1.02	(0.91 - 0.94)	1.04	(0.93 - 1.17)
Environmental Demands	**1.15**	**(1.03 - 1.28)**	1.05	(0.94 - 1.17)	1.01	(0.90 - 1.13)
Working Time Location	0.97	(0.91 - 1.04)	0.97	(0.91 - 1.04)	0.96	(0.90 - 1.03)

Note: Statistically significant values in bold.

Abbreviations: CI, Confidence Interval; OR, Odds Ratio

[1] Adjusted by sex, type of employment, education, age

[2] Adjusted by sex, type of employment, education, age, dietary pattern, sport, unfavorable cholesterol, hypertension, BMI, diabetes mellitus, smoking

* Imputed covariates via multiple imputations with chained equations (MICEs)
